# The Impairment of Macrophage-to-Feces Reverse Cholesterol Transport during Inflammation Does Not Depend on Serum Amyloid A

**DOI:** 10.1155/2013/283486

**Published:** 2013-01-30

**Authors:** Maria C. de Beer, Joanne M. Wroblewski, Victoria P. Noffsinger, Ailing Ji, Jason M. Meyer, Deneys R. van der Westhuyzen, Frederick C. de Beer, Nancy R. Webb

**Affiliations:** ^1^Saha Cardiovascular Research Center, University of Kentucky Medical Center, Lexington, KY 40536, USA; ^2^Department of Physiology, University of Kentucky Medical Center, Lexington, KY 40536, USA; ^3^Department of Internal Medicine, University of Kentucky Medical Center, Lexington, KY 40536, USA; ^4^Department of Molecular and Cellular Biochemistry, University of Kentucky Medical Center, Lexington, KY 40536, USA; ^5^Department of Veterans Affairs Medical Center, Lexington, KY 40511, USA

## Abstract

Studies suggest that inflammation impairs reverse cholesterol transport (RCT). We investigated whether serum amyloid A (SAA) contributes to this impairment using an established macrophage-to-feces RCT model. Wild-type (WT) mice and mice deficient in SAA1.1 and SAA2.1 (SAAKO) were injected intraperitoneally with ^3^H-cholesterol-labeled J774 macrophages 4 hr after administration of LPS or buffered saline. ^3^H-cholesterol in plasma 4 hr after macrophage injection was significantly reduced in both WT and SAAKO mice injected with LPS, but this was not associated with a reduced capacity of serum from LPS-injected mice to promote macrophage cholesterol efflux *in vitro*. Hepatic accumulation of ^3^H-cholesterol was unaltered in either WT or SAAKO mice by LPS treatment. Radioactivity present in bile and feces of LPS-injected WT mice 24 hr after macrophage injection was reduced by 36% (*P* < 0.05) and 80% (*P* < 0.001), respectively. In contrast, in SAAKO mice, LPS did not significantly reduce macrophage-derived ^3^H-cholesterol in bile, and fecal excretion was reduced by only 45% (*P* < 0.05). Injection of cholesterol-loaded allogeneic J774 cells, but not syngeneic bone-marrow-derived macrophages, transiently induced SAA in C57BL/6 mice. Our study confirms reports that acute inflammation impairs steps in the RCT pathway and establishes that SAA plays only a minor role in this impairment.

## 1. Introduction

Epidemiological studies have identified a strong inverse relationship between the risk of cardiovascular disease and plasma HDL levels [1]. One of the primary antiatherogenic properties of HDL is thought to be its role in reverse cholesterol transport (RCT), a process whereby excess cholesterol is removed from peripheral tissues, including lipid-loaded macrophages in the vessel wall, and transported to the liver for excretion in the bile and feces [2]. Accelerated atherosclerosis has been associated with inflammatory diseases such as rheumatoid arthritis [3], and inflammatory biomarkers are increasingly used as predictors of cardiovascular disease progression [4, 5]. Inflammation gives rise to numerous metabolic and structural changes in lipoproteins, particularly HDL, which may impact the ability of HDL to mediate RCT [6, 7]. Inflammatory HDL undergoes structural and compositional changes, most notably an increase in its serum amyloid A (SAA) content, to the extent that SAA can become the major apolipoprotein of HDL [8]. SAA is a major hepatic acute phase reactant and can account for as much as 2.5% of the protein produced in the liver during severe inflammation [9]. During the acute phase, plasma SAA concentrations rise rapidly with peak concentrations exceeding 1 mg/mL. Approximately 95% of plasma SAA is associated with HDL [8]. 

Two major acute phase SAA isoforms are expressed in mice, SAA1.1 and SAA2.1, which are synthesized in the liver upon inflammatory cytokine stimulation [10]. Other members of the murine SAA family include the constitutively expressed SAA4 [11], which is a minor HDL protein, and SAA3, which is primarily expressed extrahepatically, but does not comprise a major protein of acute phase HDL [12]. Studies on the impact of SAA on HDL metabolism during inflammation are confounded by the fact that the inflammatory mediators that induce SAA also impact numerous other metabolic systems, so that ascribing specific functions to SAA becomes challenging. To delineate the specific impact of acute phase SAAs on HDL metabolism and function, we recently generated gene-targeted mice lacking both SAA1.1 and SAA2.1 (SAAKO mice) [13].

According to previous reports, macrophage RCT is impaired in mice during an acute phase inflammatory response induced by LPS [14, 15] or zymosan [16]. In this study, we determined the extent to which acute phase SAA1.1 and SAA2.1 contribute to this impairment by quantifying *in vivo* macrophage RCT in wild-type (WT) and SAAKO mice in the presence or absence of LPS-elicited endotoxemia. Our results support the conclusion that inflammation impairs macrophage-to-feces RCT and that acute phase SAA has little impact on this impairment. We also report that administration of cholesterol-loaded J774 macrophages, a commonly used procedure in macrophage-to-feces RCT studies, independently induces a transient acute phase response in mice.

## 2. Materials and Methods

### 2.1. Animals

C57BL/6 mice were obtained from Jackson Laboratories. Mice lacking SAA1.1 and SAA2.1 were generated by targeted deletion of both mouse acute phase SAA genes *SAA1* and *SAA2* (InGenious Targeting Laboratory Inc., Stony Brook, NY) using embryonic stem cells derived from C57BL/6 ×129 SVEV mice as described previously [13]. Mice were maintained in a pathogen-free facility under equal light-dark cycles with free access to water and food. All procedures were carried out in accordance with PHS policy and approved by the Lexington Kentucky Veterans Affairs Medical Center Institutional Animal Care and Use Committee (Assurance number A3506-01).

### 2.2. Cell Culture

#### 2.2.1. J774 Cells

J774 macrophages were kindly provided by Dr. G.H. Rothblat (University of Pennsylvania) and maintained in RPMI-1640 supplemented with 10% heat-inactivated FBS and 50 *μ*g/mL gentamicin. For *in vivo* RCT experiments, cells were grown in suspension in HEPES-buffered RPMI-1640 containing 10% heat-inactivated FBS and 50 *μ*g/mL gentamicin. The cells were cholesterol loaded for 24 hr in HEPES-buffered RPMI-1640 containing 1% FBS, 25 *μ*g/mL acetylated LDL (acLDL), and 5 *μ*Ci/mL ^3^H-cholesterol (35–50 Ci/mmol, Amersham Biosciences). On the day of injection, cells were washed, equilibrated for 2 hr in HEPES-buffered RPMI-1640 containing 0.2% fatty acid free BSA, centrifuged, and resuspended in minimal essential media (MEM) prior to injection into mice (2.5 × 10^6^ cells in 0.5 mL). For some studies, J774 cells were prepared for intraperitoneal injection into mice as described above, except that ^3^H-cholesterol was omitted from the media during the cholesterol loading step. 

#### 2.2.2. Bone-Marrow-Derived Macrophages (BMMs)

BMMs were harvested and cultured as described [17]. Briefly, BMMs were harvested from the tibias and femurs of C57BL/6 mice 5 days after intraperitoneal injection of 1 mL 2% biogel beads [18] by flushing the bone cavities with PBS. Bone-marrow was homogenized by drawing through an 18G needle and was maintained in sterile nontissue culture treated flasks using RPMI-1640 supplemented with 1% P/S, 10% FBS, and 15% L-cell-conditioned medium. The medium was changed on days 3 and 5, and on day 6 BMM were treated for 24 hr with RPMI 1640 containing 1% FBS, 1% P/S, and 25 *μ*g/mL acLDL. On day 7 cells were equilibrated for 2 hr in RPMI-1640 containing 0.2% fatty acid-free BSA, dislodged from the tissue culture flask with nonenzymatic cell dissociation buffer (Sigma), centrifuged, and suspended in MEM prior to intraperitoneal injection of 2.5 × 10^6^ cells/0.5 mL. 

#### 2.2.3. Rat Fu5AH Hepatoma Cells

Fu5AH cells, kindly provided by Dr. G.H. Rothblat (University of Pennsylvania), were maintained in MEM supplemented with 5% FBS and 50 *μ*g/mL gentamicin.

### 2.3. Semiquantitative Real-Time PCR (RT-PCR)

RT-PCR was performed as described previously [13]. Briefly, total RNA was isolated from mouse liver using the TRIzol Reagent (Molecular Research Center), and 10 *μ*g was treated with TURBO DNA-Free DNAse (Ambion) according to the manufacturer's protocol. cDNA synthesis was performed using 500 ng RNA and the Applied Biosystems High Capacity cDNA kit. Amplification was carried out using the iCycler IQ5 system (Bio-Rad). Quantification was performed in duplicate using the standard curve method and normalized to GAPDH. The primers used are as follows: **mSRBI**, NM_016741:forward 5′-CTTCATGACACCCGAATCCT-3′, Reverse 5′-AATGCCTTCAAACACCCTTG-3′, 114bp; **mCyp7A1**, NM_007824 forward 5′-AGCAACTAAACAACCTGCCAGTACTA-3′, reverse 5′-GTCCGGATATTCAAGGATGCA-3′, 84bp; **mABCA1**, NM_013454: forward 5′-AGCCAGAAGGGAGTGTCAGA-3′, reverse 5′-CATGCCATCTCGGTAAACCT-3′ 102bp; **mABCG1**, NM_ 009593: forward 5′-AGGCCTACTACCTGGCAAAGA-3′, reverse 5′-GCAGTAGGCCACAGGGAACA-3′, 68bp; **mABCG5**, NM_031884: forward 5′-TGGATCCAACACCTCTATGCTAAA-3′, reverse 5′-GGCAGGTTTTCTCGATGAACTG-3′ 77bp; **mABCG8**, NM_026180: forward 5′-TGCCCACCTTCCACATGTC-3′, reverse 5′-ATGAAGCCGGCAGTAAGGTAGA-3′ 73bp; **mABCB11**, NM_021022: forward 5′-AAGCTACATCTGCCTTAGACACAGAAA-3′, reverse 5′-CAATACAGGTCCGACCCTCTCT-3′, 84bp.

### 2.4. *In Vivo* Macrophage RCT

RCT studies were carried out in accordance with a well-established model [14, 19]. Three separate experiments were performed in ~16-week-old female SAAKO and WT mice (*n* = 4-5 per group per experiment) that were housed in individual cages and fed a normal rodent diet *ad libitum*. Anesthetized mice received a subcutaneous injection of 0.8 *μ*g/g body weight lipopolysaccharide (LPS; from *E. coli* 0111:B4, Sigma L 2630) in ~100 *μ*L PBS or an injection of PBS alone. After 4 hr, mice were administered intraperitoneally 2.5 × 10^6^ radiolabeled J774 macrophages (2.78–3.87 × 10^6^ dpm/mouse) prepared as described above. Blood was collected via retroorbital bleed 4 hr after LPS administration and just prior to injection of macrophages and then again 4 hr after macrophage administration. Feces were collected throughout the course of the study. Mice were euthanized 24 hr after macrophage administration, and blood, bile, and liver were collected. To study whether intraperitoneal injection of macrophages induces an acute phase response in mice, BMM and J774 cells were prepared as described above, except that ^3^H-cholesterol was omitted from the media during the cholesterol loading step. 

### 2.5. *Ex Vivo* Macrophage Cholesterol Efflux Experiments

For efflux assays, plasma was obtained from WT and SAAKO mice 8 hr after subcutaneous injection of PBS or LPS (0.8 *μ*g/g). Mouse serum was prepared as described [20]. Cellular cholesterol efflux experiments were carried out essentially as described [21, 22]. Briefly, J774 cells (~60% confluent) in 12-well plates were labeled for 48 hr with 0.2 *μ*Ci/mL [^3^H]cholesterol (35–50 Ci/mmol, Amersham Biosciences) in RPMI-1640 supplemented with 10% heat-inactivated FBS, 50 *μ*g/mL gentamicin, and 50 *μ*g/mL acLDL. Cells were then washed three times with PBS containing 1 mg/mL BSA (PBS-BSA) and equilibrated overnight in RPMI-1640 containing 0.2% fatty acid-free BSA (RPMI-BSA). Following two additional washes with PBS-BSA, cells were harvested to determine total dpm incorporated (time zero). Efflux to RPMI-BSA with or without 2.5% serum from control or LPS-treated mice was measured over 5 hr at 37°C. Following the incubation, cell media was collected and centrifuged to remove detached cells. Radioactivity in the medium was measured directly in a Packard *β* liquid scintillation counter. Adherent cells were washed at 4°C twice with PBS-BSA and twice with PBS and then solubilized in 0.1 N NaOH and counted for radioactivity. Efflux of cellular [^3^H]-cholesterol was expressed as the percentage of the total radioactivity incorporated into cells at time zero that was present in the media. 

### 2.6. Cholesterol Influx into Hepatoma Cells

Fu5AH hepatoma cells, maintained as described above, were seeded into 12-well plates. When cultures were more than 90% confluent, cells were pretreated for 1 hr with 10 *μ*M block lipid transport-1 (BLT-1) in MEM containing 0.5% FAF-BSA, to inhibit SR-BI. Control cells were treated with the DMSO vehicle. Cells were subsequently incubated, in the presence or absence of BLT-1, in MEM alpha supplemented with 5% serum obtained from mice at the termination of RCT experiments (i.e., 24 hr after administration of [^3^H]-cholesterol-labeled macrophages) to monitor the uptake of [^3^H]-cholesterol which includes both free and esterified [^3^H]-cholesterol. After 6 hr, cells were washed at 4°C, 4 times with PBS containing 0.2% fatty acid-free BSA and twice with PBS, solubilized in 0.1 N NaOH, and counted for radioactivity. 

### 2.7. Western Blot Analyses

Total liver lysates and liver membrane protein extracts were prepared for western blot analyses essentially as described [23]. Briefly ~100 mg liver was homogenized with an Ultra Turrax T8 probe homogenizer in 1.2 mL 20 mM Tris pH 7.5, 2 mM MgCl_2_, and 0.25 M sucrose containing protease inhibitors. After centrifugation at 12000 ×g for 10 min at 4°C, the supernatant (i.e., total liver lysate) was used for western blot analyses. Alternatively, membrane proteins were isolated by centrifuging the total liver lysate at 100,000 ×g for 30 min at 4°C; the membrane protein pellet was resuspended in the homogenization buffer described above. Protein concentrations of liver lysates and membrane protein suspensions were determined by BCA assay (Pierce). Liver proteins were size-fractionated by SDS-PAGE and immunoblotted with the following antibodies: anti-human/mouse SR-BI (1 : 1000) [24], anti-mouse/human ABCG1 (1 : 500; Novus NB400-132), anti-ABCG5 (1 : 10,000; a generous gift from Dr. Gregory Graf, University of Kentucky). For loading controls liver lysates/membranes were immunoblotted with anti-*β* actin (1 : 2000; Sigma, A5441) or anti-mouse calnexin (1 : 2000; Enzo Life Sciences). For western blot analyses of plasma proteins, aliquots of plasma (0.3–0.5 *μ*L) were subjected to SDS-PAGE and immunoblotted using rabbit anti-mouse SAA (De Beer laboratory) or rabbit anti-mouse SAP (gift from Dr. Mark Pepys, University College of London, UK). Plasma SAA concentrations were determined by quantitative immunoblotting using acute phase mouse HDL with known SAA content as a standard. 

### 2.8. Endotoxin Assay

The Limulus Amebocyte Lysate kit (Genscript cat. no. L00351) was used to verify that plasma/serum samples used for *ex vivo* efflux or influx assays contained less than 0.005 EU/mL of endotoxin.

### 2.9. Statistical Analyses

Data are expressed as the mean ± SEM. Results were analyzed by two-way ANOVA with Bonferroni posttest. Significance was defined as *: *P* < 0.05; **: *P* < 0.01; ***: *P* < 0.001.

## 3. Results

### 3.1. Mice Deficient in SAA1.1 and SAA2.1 Mount a Normal LPS-Induced Acute Phase Response

**Figure 1:**
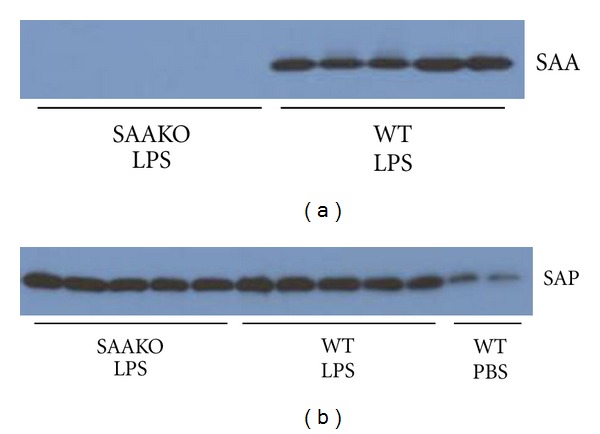
SAAKO mice are capable of mounting an acute phase response. WT and SAAKO mice were injected subcutaneously with 0.8 *μ*g/g LPS followed 4 hr later by an intraperitoneal injection of ^3^H-cholesterol-labeled J774 macrophages. Control WT mice received PBS only. Aliquots of plasma collected from individual mice 24 hr after macrophage administration (28 hr after LPS) were subjected to SDS-PAGE and immunoblotted using (a) rabbit anti-mouse SAA; and (b) rabbit anti-mouse SAP.

According to previous reports, macrophage-to-feces RCT is reduced in mice during an LPS-elicited acute phase response [14, 15]. To investigate whether SAA contributes to this impairment, we carried out studies using our recently developed gene-targeted mice that lack both major acute phase SAA isoforms, SAA1.1 and SAA2.1 [13]. In 3 separate studies, the movement of ^3^H-cholesterol from macrophages to feces was monitored in WT and SAAKO mice under both normal and inflammatory conditions using an established RCT model [14, 19] (*n* = 4-5 per group per experiment). An acute phase response was evoked in mice by injecting a relatively modest dose of LPS subcutaneously (0.8 *μ*g/g body weight). As expected, plasma SAA was readily detected in WT but not SAAKO mice 28 hr after LPS injection (Figure 1(a)), reaching values of ~130 *μ*g/mL in the WT mice. The ability of SAAKO mice to mount an acute phase response was demonstrated by the marked induction of serum amyloid P component (SAP), which was present in the plasma at comparable levels in WT and SAAKO mice 28 hr after LPS injection (Figure 1(b)). 

### 3.2. SAA Is Not Required for the Reduction in Macrophage to Plasma RCT during Endotoxemia

**Figure 2:**
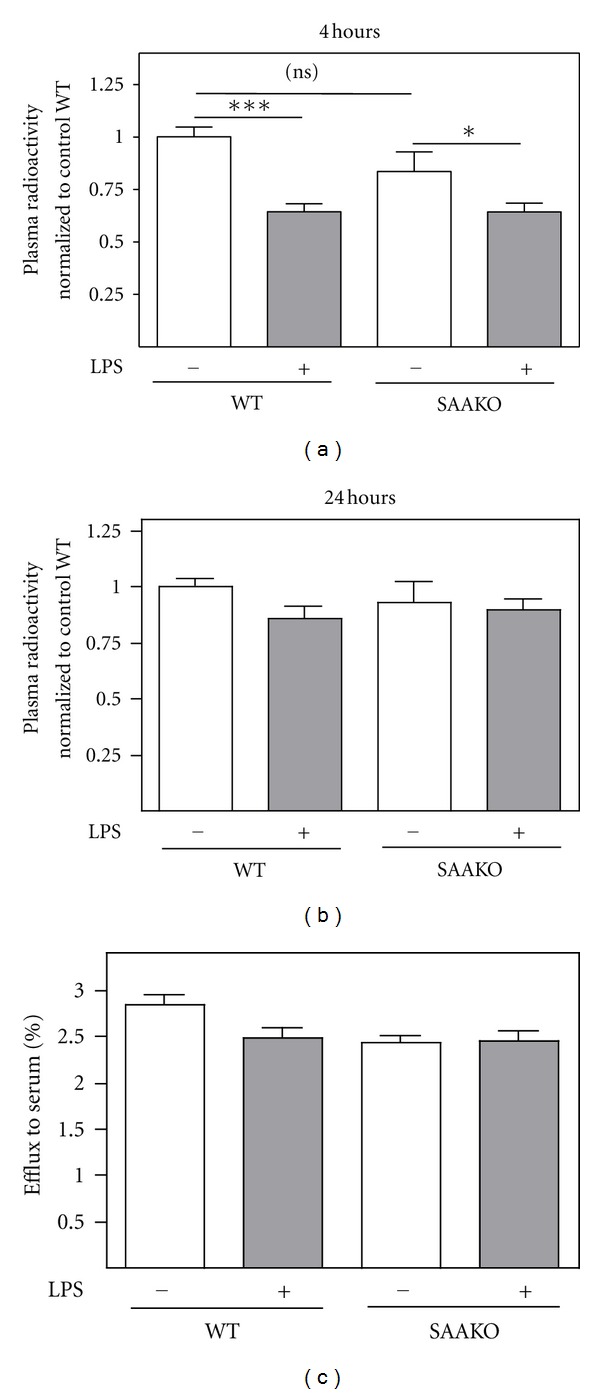
SAA does not contribute to reduced macrophage to plasma RCT during an acute phase response. WT and SAAKO mice were injected subcutaneously with PBS or 0.8 *μ*g/g LPS as indicated, followed by an intraperitoneal injection of ^3^H-cholesterol-labeled J774 macrophages 4 hr later. Radioactivity in plasma was determined 4 hr (a) and 24 hr (b) after macrophage injection (8 hr and 28 hr after LPS injection). The data shown in (a) and (b) were compiled from 3 separate experiments (*n* = 4-5 mice per group per experiment) after normalization to the PBS-injected WT mice. For the 3 experiments, radioactivity recovered in plasma of control WT mice at 4 and 24 hr ranged from 1.0% to 2.4% and 2.0% to 5.0% of the injected radioactivity, respectively. (c) Cholesterol efflux assays were carried out using J774 cells as described in Materials and Methods using serum (2.5% v : v) collected 8 hr after administration of LPS (0.8 *μ*g/g body weight; *n* = 5). Values are the mean ± SEM; *P < 0.05, ***P < 0.001.

For the assessment of *in vivo* macrophage RCT, mice were injected intraperitoneally with ^3^H-cholesterol-labeled macrophages 4 hr after administration of LPS or PBS control. The initial step of RCT (i.e., the movement of ^3^H-cholesterol from macrophages to the plasma compartment) was monitored by quantifying plasma radioactivity 4 hr and 24 hr after macrophage injection (8 hr and 28 hr after LPS injection). In agreement with previous studies [14, 15], the amount of macrophage-derived ^3^H-cholesterol present in plasma was lower in WT mice undergoing an acute phase response compared to control WT mice, an effect that was statistically significant at the earlier time point (Figure 2(a)). Similarly, there was significantly decreased radioactivity in plasma of LPS-injected SAAKO mice compared to control SAAKO mice when measured at 4 hr (Figure 2(a)). At 24 hr, the amount of radioactive tracer in plasma was similar for all four groups of mice (Figure 2(b)). To more directly assess whether SAA impacts macrophage cholesterol efflux, we carried out *in vitro* cholesterol efflux assays using cholesterol-loaded J774 cells and serum collected from WT and SAAKO mice 8 hr after LPS or saline injection (Figure 2(c)). Results from these assays indicated that the reduced amount of macrophage-derived ^3^H-cholesterol in plasma of LPS-injected mice was not associated with a reduction in the capacity of acute phase serum from either WT or SAAKO mice to promote cholesterol efflux.

### 3.3. Hepatic Accumulation of Macrophage-Derived Cholesterol Is Not Altered during Endotoxemia, Regardless of the Presence of SAA

**Figure 3:**
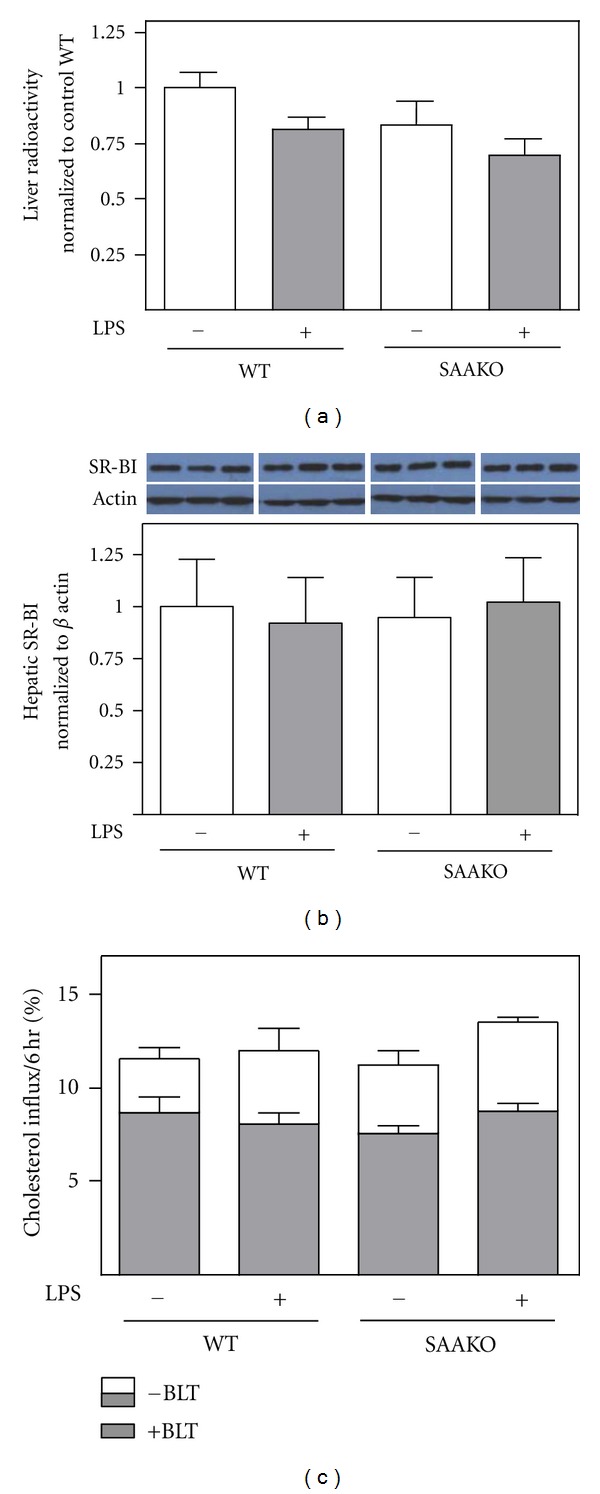
Endotoxemia does not alter hepatic accumulation of macrophage-derived ^3^H-cholesterol, regardless of the presence of SAA. WT and SAAKO mice were injected subcutaneously with PBS or 0.8 *μ*g/g LPS as indicated, followed by intraperitoneal injection of ^3^H-cholesterol-labeled J774 macrophages 4 hr later. (a) Radioactivity in liver was determined 24 hr after macrophage injection (28 hr after LPS injection). The data shown were compiled from 3 separate experiments (*n* = 4-5 per group per experiment) after normalization to PBS-injected WT mice. Values are the mean ± SEM. Liver counts for PBS-injected WT and SAAKO mice were similar, ranging from 3.9% to 8.5% of the injected tracer for the 3 experiments. (b) SR-BI in total liver lysates (10 *μ*g protein) was detected by immunoblotting and quantified by densitometry. Results from each of the 3 experiments (*n* = 4-5 per group per experiment) were expressed relative to PBS-injected WT mice after normalization to *β*-actin. A representative western blot is shown. (c) Fu5AH hepatoma cells were preincubated for 1 hr with or without 10 *μ*M BLT-1 and then for 6 hr with media ± BLT-1 supplemented with 5% serum obtained from mice 24 hr after administration of ^3^H-cholesterol-labeled macrophages (28 hr after LPS injection). The amount of radioactivity that was taken up by cells in the presence of BLT-1 is indicated by the shaded portion of the bars.

We next investigated whether hepatic accumulation of macrophage-derived ^3^H-cholesterol is altered in WT or SAAKO mice during an acute inflammatory response. The amount of radioactivity in livers of LPS-injected WT and SAAKO mice 24 hr after macrophage injection was modestly lower compared to the corresponding control mice, but this difference did not reach statistical significance in either strain (Figure 3(a)). According to some [14] but not all [15] reports, endotoxemia results in transcriptional downregulation of SR-BI, an important HDL receptor in the liver that mediates selective lipid uptake from HDL. Our data indicate that SR-BI mRNA abundance was significantly reduced in mouse livers 28 hr after LPS injection, and this downregulation was not dependent on SAA (Table 1). However, SR-BI protein levels were not significantly altered in either strain by LPS injection (Figure 3(b)). Since alterations in HDL-C uptake by the liver during endotoxemia may not necessarily be evident in static measures of hepatic ^3^H-cholesterol content 24 hr following radiolabeled macrophage injection, we investigated whether there were any differences in uptake of the radioactive tracer from the sera of control or LPS-injected WT and SAAKO mice by Fu5AH hepatoma cells [22, 25]. Uptake studies were carried out in the presence and absence of 10 *μ*M BLT-1, which has been shown to block SR-BI-mediated selective lipid uptake from HDL [26]. For all of the serum samples assayed, BLT-1 reduced the uptake of the radioactive tracer ~30% (Figure 3(c)), suggesting that only a portion of hepatic uptake of the macrophage-derived radioactive tracer was mediated by SR-BI. Furthermore, our data indicated no significant differences in the uptake of the radioactive tracer from serum collected from control or LPS-injected mice, regardless of the presence of SAA (Figure 3(c)). 

### 3.4. SAA Has Limited Impact on Impaired Macrophage to Bile and Feces RCT during Endotoxemia

**Figure 4:**
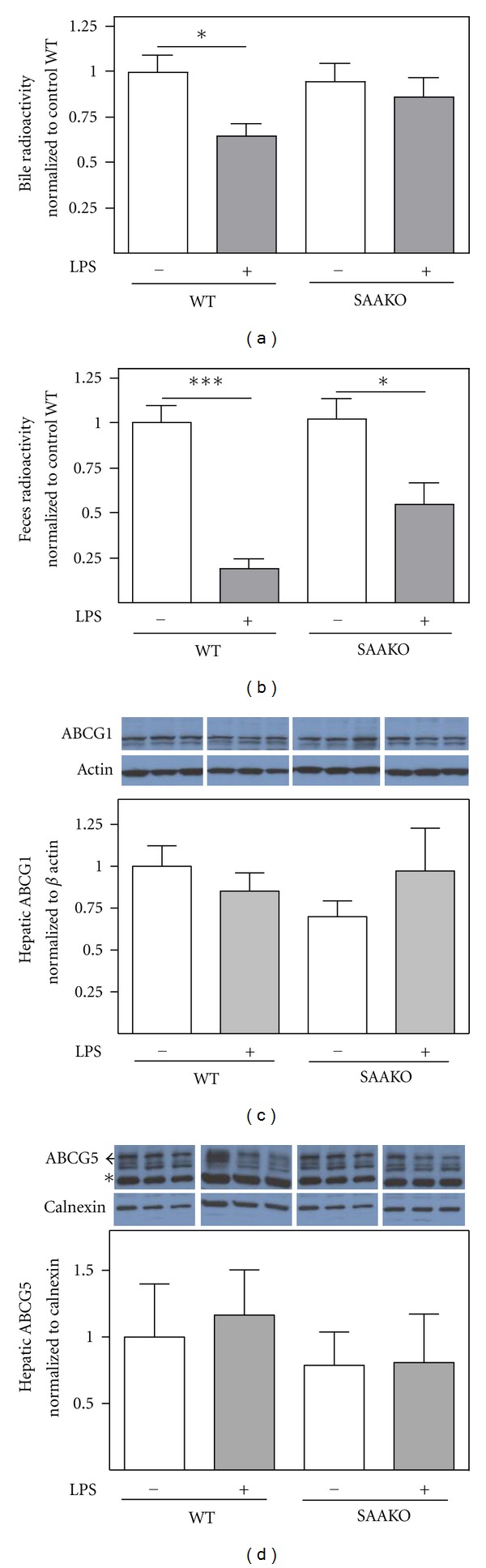
SAA has little impact on the impairment in biliary and fecal excretion of macrophage-derived ^3^H-cholesterol during endotoxemia. WT and SAAKO mice were injected subcutaneously with PBS or 0.8 *μ*g/g LPS as indicated, followed 4 hr later by intraperitoneal injection of ^3^H-cholesterol-labeled J774 macrophages. (a) Radioactivity in bile was determined 24 hr after macrophage injection. The data shown are compiled from 3 separate experiments (*n* = 4-5 per group per experiment) and normalized to the PBS-injected WT mice. Values are the mean ± SEM; *P < 0.05%. For each of the 3 experiments, the amount of radioactivity recovered in the bile of PBS-injected WT and SAAKO mice was similar, ranging from 0.01% to 0.04% of injected tracer per *μ*L of bile. (b) Feces were collected during the 24 hr RCT experiments, and the total amount of radioactivity was determined. Due to methodological issues in one of the experiments, data from only 2 experiments were compiled (*n* = 5 per group per experiment) and normalized to the PBS-injected WT mice. Values are the mean ± SEM; ***P < 0.001; *P < 0.05. For each of the 2 experiments, the amount of radioactivity recovered in feces of PBS-injected WT and SAAKO mice was similar, ranging from 1.8%–2.6% of the injected tracer. (c) ABCG1 in liver lysates (40 *μ*g protein) was identified by immunoblotting and quantified by densitometry. Results from each of the 3 experiments (*n* = 4-5 per group per experiment) were expressed relative to PBS-injected WT mice after normalization to *β*-actin. A representative western blot is shown. (d) ABCG5 in liver membranes (50 *μ*g protein) was detected by immunoblotting and quantified by densitometry. Results from each of the 3 experiments (*n* = 4-5 per group per experiment) were expressed relative to PBS-injected WT mice after normalization to calnexin. A representative western blot is shown, with the immature (reticular) and mature (post-Golgi) forms of ABCG5 indicated with a *carat*. The band indicated by an *asterisk *is observed in G5G8-deficient mice suggesting that it represents a nonspecific signal [23].

Endotoxemia significantly impaired biliary and fecal excretion of macrophage-derived ^3^H-cholesterol in WT mice (Figures 4(a) and 4(b)), in line with previously published data [14, 15]. At 24 hr after macrophage injection, the amount of radioactivity present in the bile and feces of LPS-injected WT mice was reduced 36% and 80%, respectively, compared to control WT mice. On the other hand, endotoxemia did not significantly impact the amount of macrophage-derived ^3^H-cholesterol present in bile of SAAKO mice, and fecal excretion was reduced by only 45% (Figures 4(a) and 4(b)). Thus, our results confirm previous findings that macrophage-to-feces RCT is significantly reduced in mice undergoing an acute phase response and establishes that SAA has limited impact on this impairment. 

### 3.5. Deficiency of SAA Does Not Impact the Effect of Inflammation on Hepatic Expression of Enzymes and Transporters Involved in Cholesterol Flux

Previous studies have established that hepatic expression of genes involved in cholesterol transport and biliary excretion is altered in mice undergoing an acute phase response [14, 15]. In our studies, a moderate dose of LPS had no effect on hepatic expression of ABCA1 or Cyp7A1 mRNA (Table 1) in either WT or SAAKO mice when assessed 28 hr after LPS injection. On the other hand, ABCG1, ABCG5, ABCG8, and ABCB11 expression were all significantly suppressed after LPS treatment, and this suppression was not impacted by SAA deficiency (Table 1). There was no evidence that endotoxemia altered hepatic ABCG1 or ABCG5 protein in WT or SAAKO mice (Figures 4(c) and 4(d)). 

### 3.6. Peritoneal Injection of Cholesterol-Loaded J774 Cells Induces a Transient Inflammatory Response in C57BL/6 Mice

**Figure 5:**
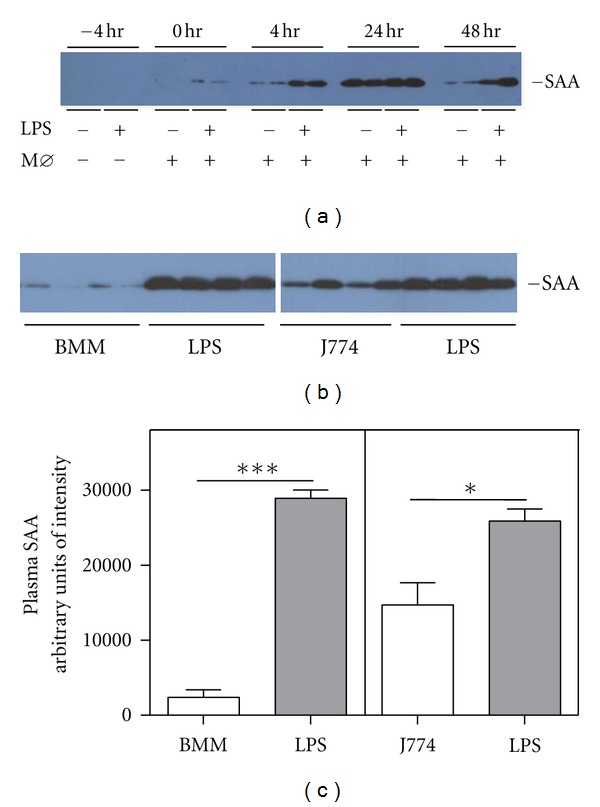
Intraperitoneal injection of J774 cells induces an acute phase response in C57BL/6 mice. (a) WT mice were injected subcutaneously with PBS or 0.8 *μ*g/g LPS (−4 hr) followed 4 hr later by an intraperitoneal injection of ^3^H-cholesterol-labeled J774 macrophages (0 hr); *n* = 2 per treatment). Aliquots of plasma (0.25 *μ*L) collected from individual mice at the indicated times were subjected to SDS-PAGE, followed by immunoblotting for SAA. (b) In two parallel studies, C57BL/6 mice were injected subcutaneously with PBS or 0.8 *μ*g/g LPS, and 4 hr later, the PBS-injected mice were injected *i.p*. with cholesterol-loaded J774 macrophages or C57BL/6 BMM, as indicated (*n* = 4 per treatment). Aliquots of plasma (0.25 *μ*L) collected from individual mice 28 hr after PBS/LPS injection (i.e., 24 hr after macrophage injection) were subjected to SDS-PAGE, followed by immunoblotting for SAA. (c) Relative plasma SAA concentrations 28 hr after PBS/LPS injection (i.e., 24 hr after macrophage injection) as determined by densitometry (*n* = 4). Values are the mean ± SEM; ***P < 0.001; *P < 0.05.

In the course of our *in vivo* RCT experiments we routinely monitored the extent of induction of inflammation in LPS-injected mice. Since the SAAKO mice do not express the major mouse acute phase isoforms SAA1.1 and SAA2.1, we assessed the expression of serum amyloid P component (SAP), another major acute phase reactant in mice. Unexpectedly, we readily detected SAP in the plasma of all mice injected with J774 macrophages, irrespective of LPS administration (data not shown). This finding prompted us to investigate the possibility that the experimental protocol itself produced an inflammatory response in mice. To this end, J774 macrophage foam cells were prepared according to the protocol for RCT and injected into WT mice 4 hr after administration of either PBS or LPS (“0 hr” on Figure 5(a)), and plasma samples were collected at selected intervals during the course of the experiment and immunoblotted for SAA. As expected, LPS elicited a robust inflammatory response, as evidenced by a marked increase in plasma SAA that was detectable 4 hr after LPS injection and persisted for at least 48 hr (Figure 5(a)). Notably, intraperitoneal administration of J774 macrophages also evoked a robust inflammatory response that was detectable 4 hr after the injection of the cells, increased by 24 hr, and was still evident at 48 hr after administration (Figure 5(a)). This finding suggested that intraperitoneal administration of allogeneic macrophages may promote an inflammatory response in mice (J774 cells were originally derived from the BALB/c strain). To investigate this possibility, we carried out parallel studies in which C57BL/6 mice were injected with either LPS, J774 cells, or syngeneic bone-marrow-derived macrophages. BMMs from C57BL/6 mice and J774 macrophages were converted to foam cells according to the protocol for RCT experiments, except that ^3^H-cholesterol was omitted during cholesterol loading. The cells were then injected into C57BL/6 mice 4 hr after administration of either PBS or LPS, and plasma samples were immunoblotted for SAA 24 hr after injection of cells (Figure 5(b)), a time when SAA was markedly induced in the previous study (Figure 5(a)). Whereas injection of J774 cells elicited a marked increase in plasma SAA (corresponding to ~57% of the level evoked by a moderate dose (0.8 *μ*g/g body weight) of LPS; Figure 5(c)), SAA was virtually undetectable in mouse plasma 24 hr after BMM injection, corresponding to ~8% of the amount elicited by LPS. 

## 4. Discussion

Based on a number of large population studies, it is recognized that plasma levels of HDL and its major apolipoprotein apoA-I are inversely correlated with the risk of atherosclerosis. One of the most widely accepted mechanisms to explain HDL's cardioprotective effect is its role in RCT, whereby HDL promotes the removal of excess cholesterol from peripheral cells, including macrophage foam cells in the vessel wall, and delivery of cholesterol to the liver for excretion. The RCT pathway involves several steps: (1) mobilization of cellular cholesterol and efflux to HDL or HDL-derived lipid-poor apolipoproteins; (2) esterification of free cholesterol in HDL to form cholesteryl ester (CE) by lecithin-cholesterol acyltransferase; (3) receptor-mediated uptake of HDL-CE by hepatocytes, either selectively by SR-BI or via whole particle uptake through a poorly defined pathway; and (4) deesterification and excretion of cholesterol into bile, either in the form of free cholesterol or as bile acids. In species that express cholesterol ester transfer protein (CETP), including humans but not mice, an alternate pathway for RCT is through the transfer of HDL-CE to apoB-containing lipoproteins and their subsequent uptake into the liver. The extent to which inflammation impacts the capacity of HDL to participate in RCT has significant clinical relevance, given the increasing incidence of chronic inflammatory conditions in humans, including rheumatoid arthritis, type 2 diabetes; and the metabolic syndrome; all of which are associated with an increased risk for atherosclerotic cardiovascular disease. Data from this study agrees with earlier reports [14–16] that acute inflammation impairs steps in the RCT pathway when assessed in an established macrophage-to-feces mouse model and determines that acute phase SAA has little impact on this impairment. We also determined that in the established RCT model, injection of J744 macrophages results in a transient inflammatory response.

In our studies, LPS-induced endotoxemia resulted in an ~35% decrease in the movement of ^3^H-cholesterol from macrophages to the plasma compartment when assessed 4 hr after macrophage injection. This result is consistent with previous reports that endotoxemia acutely impairs macrophage to plasma RCT and supports the conclusion that the extent of the impairment is related to the magnitude of the acute phase response [14]. McGillicuddy reported that while a relatively low dose of LPS (0.3 mg/kg *s.c.*) did not reduce ^3^H-cholesterol counts in plasma at any time point after macrophage injection, a high dose of LPS (3 mg/kg *s.c.*) resulted in a significant ~35% and ~20% decline in macrophage to plasma RCT when measured at 4 hr and 24 hr, respectively. In a study by another group, a similar dose of LPS administered *i.p*. resulted in a 33% and 27% reduction in the movement of macrophage-derived cholesterol to the plasma at 6 and 24 hr, respectively [15]. On the other hand, the moderate dose of LPS used in our study (0.8 mg/kg *s.c.*) produced an effect that appeared to be more transient, since a significant difference in the amount of macrophage-derived cholesterol in plasma of control and LPS-injected mice was detected 4 hr, but not 24 hr, after macrophage injection. Notably, SAA deficiency did not alter the acute effect of endotoxemia to reduce macrophage to plasma RCT, suggesting that the duration of this impairment is not due to differences in the magnitude of the induction of SAA at different doses of LPS. 

Previous studies have investigated whether SAA impacts the ability of HDL to carry out individual steps in the RCT pathway, including cellular cholesterol efflux. Studies investigating the impact of inflammation and SAA per se on cholesterol efflux have been conflicting, depending on the nature of the HDL and the cell system used in the assay. In several reports, SAA, either associated with HDL or in a lipid-free form, was shown to promote cholesterol efflux through both ABCA1-dependent and ABCA1-independent mechanisms [27–33]. With regards to ABCG1-dependent efflux, our group reported that inflammatory remodeling of mouse HDL leads to an increase in its capacity to promote efflux, but the presence of SAA did not play a role in this enhancing effect [33]. On the other hand, in two recent studies, SAA-enriched HDL from human subjects undergoing acute sepsis [15] or HDLs isolated from humans or mice subjected to experimental endotoxemia [14] showed a reduced capacity to promote macrophage cholesterol efflux. Using an overexpression approach, Annema et al. concluded that SAA does not alter the rate of movement of ^3^H-cholesterol from macrophages to mouse plasma *in vivo* in the absence of an acute phase response [15]. Results from the current study suggest that serum from mice collected 8 hr after LPS injection is not altered in its capacity to stimulate efflux from cholesterol-loaded J774 macrophages compared to control serum, regardless of the presence of SAA. Thus, at least in mice, the ability of SAA to modulate macrophage cholesterol efflux appears to be minimal. 

Our data indicate that LPS-induced endotoxemia in either WT or SAAKO mice does not significantly alter macrophage to liver RCT, consistent with findings from earlier studies [14, 15]. While SR-BI mRNA expression appeared to be significantly reduced in livers of both WT and SAAKO mice 28 hr after LPS injection, SR-BI protein levels were not altered in either strain. Similarly, McGillicuddy et al. reported significantly reduced hepatic expression of SR-BI mRNA without a commensurate change in SR-BI protein 48 hr after high dose LPS injection [14]. Since hepatic accumulation of macrophage-derived ^3^H-cholesterol at a single time point is influenced in opposite directions by the rates of flux into and out of the liver, we carried out *in vitro* studies using Fu5AH hepatoma cells to determine whether hepatic uptake of ^3^H-cholesterol from acute phase plasma was significantly different compared to control and the extent to which SAA might impact such uptake. Uptake by Fu5AH cells was measured in the presence or absence of BLT-1, a known inhibitor of SR-BI-mediated selective CE uptake [26]. Our data indicated no significant difference in either SR-BI-dependent or SR-BI-independent uptake of the radioactive tracer from control or LPS-injected mouse serum, regardless of the presence of SAA. In a previous study by our group we concluded that SAA inhibits SR-BI-mediated selective lipid uptake from HDL [34]. The discrepant findings from the two studies may be due to differences in cell types used for uptake studies (Fu5AH cells versus stably transfected Chinese hamster ovary cells), the use of whole serum versus isolated HDL particles, the amount of SAA associated with the HDL fraction, and the fact that the earlier study investigated HDL enriched with SAA in the absence of inflammation.

McGillicuddy et al. [14] and Annema et al. [15] both reported that the most pronounced effect of LPS on RCT was on cholesterol flux through the liver to the bile and feces. The decrease in macrophage-to-feces RCT was associated with a significant reduction in the mRNA expression of genes involved in cholesterol transport and bile acid synthesis, including ABCG5, ABCG8, ABCB11, and CYP7A1, suggesting that one compensatory response to endotoxemia may be to inhibit the excretion of cholesterol out of the body. In our studies, the amount of macrophage-derived radiotracer present in the bile and feces of LPS-injected WT mice was reduced 36% and 80%, respectively, compared to control WT mice, confirming the previous studies. The impact of LPS on bile and feces RCT was only partially ameliorated in SAAKO mice. As reported previously [14, 15], we show that hepatic expression of ABCG5, ABCG8, and ABCB11 mRNAs is significantly reduced in WT mice 28 hr after LPS injection. However, SAA deficiency did not reverse this suppressive effect, nor did the reductions in ABCG5 mRNA coincide with reduced ABCG5 protein expression. Thus, our studies would suggest that other mechanisms in addition to effects on hepatic expression of genes involved in biliary cholesterol excretion may also play a role in the impairment of RCT during acute inflammation. As pointed out by Malik et al. [16], perturbations in macrophage-to-feces RCT after LPS injection could in part be due to non-specific metabolic effects in the liver or intestine that could have a negative impact. Whether SAA modulates the potential metabolic effects of inflammatory stimuli is the subject of ongoing studies in our laboratory. A nonbiliary, transintestinal route for neutral sterol excretion has been described [35–38], but the role of this pathway in macrophage-to-feces RCT has not been clearly delineated [39, 40]. The possibility that inflammation or SAA modulates trans-intestinal cholesterol excretion merits further study. 

An unanticipated outcome of our studies was the finding that C57BL/6 mice administered J774 macrophages undergo a transient acute phase response, as evidenced by a marked increase in plasma SAA. The inflammatory response was less severe and more short-lived than the response evoked by a modest dose of LPS. Interestingly, bone-marrow cells from syngeneic mice handled under identical conditions and with the same reagents did not produce an inflammatory response. Our conclusion that SAA has little impact on the impairment of RCT during inflammation is not negated by the finding that *i.p*. injection of J774 cells by itself leads to a modest acute phase response. Nevertheless, the use of syngeneic primary macrophages may be preferable over macrophage cell lines for *in vivo* RCT studies that are not intended to be carried out in the setting of inflammation, given the consistent finding that inflammation impairs macrophage-to-feces RCT in this commonly used surrogate model [14–16]. In summary, our findings clearly show that SAA does not substantially contribute to the impairment of RCT during inflammation.

## Figures and Tables

**Table 1:** Hepatic gene expression levels in control and LPS-treated mice.

Gene	WT		SAAKO	
	PBS	LPS	PBS	LPS
SR-BI	1.00 ± 0.06	0.42 ± 0.05***	0.77 ± 0.06	0.56 ± 0.07*
Cyp7A1	1.00 ± 0.20	0.28 ± 0.15	1.73 ± 0.35	0.94 ± 0.30
ABCA1	1.00 ± 0.06	1.22 ± 0.12	1.06 ± 0.11	1.01 ± 0.11
ABCG1	1.00 ± 0.05	0.43 ± 0.04***	0.99 ± 0.08	0.47 ± 0.06***
ABCG5	1.00 ± 0.07	0.38 ± 0.03***	1.24 ± 0.18	0.41 ± 0.04***
ABCG8	1.00 ± 0.10	0.23 ± 0.02***	1.24 ± 0.16	0.28 ± 0.04***
ABCB11	1.00 ± 0.05	0.22 ± 0.06***	0.97 ± 0.11	0.42 ± 0.09***

Values are expressed relative to control WT mice after normalization to GAPDH mRNA.

Data represent mean ± SEM, *n* = 15.

*P < 0.05, ***P < 0.001, compared to corresponding PBS-treated mice.

## References

[1] Assmann G, Gotto AM (2004). HDL cholesterol and protective factors in atherosclerosis. *Circulation*.

[2] Cuchel M, Rader DJ (2006). Macrophage reverse cholesterol transport: key to the regression of atherosclerosis?. *Circulation*.

[3] Chung CP, Oeser A, Raggi P (2005). Increased coronary-artery atherosclerosis in rheumatoid arthritis: relationship to disease duration and cardiovascular risk factors. *Arthritis and Rheumatism*.

[4] Tsimikas S, Willerson JT, Ridker PM (2006). C-reactive protein and other emerging blood biomarkers to optimize risk stratification of vulnerable patients. *Journal of the American College of Cardiology*.

[5] Rader DJ (2000). Inflammatory markers of coronary risk. *The New England Journal of Medicine*.

[6] Khovidhunkit W, Kim MS, Memon RA (2004). Effects of infection and inflammation on lipid and lipoprotein metabolism: mechanisms and consequences to the host. *Journal of Lipid Research*.

[7] Van Der Westhuyzen DR, De Beer FC, Webb NR (2007). HDL cholesterol transport during inflammation. *Current Opinion in Lipidology*.

[8] Coetzee GA, Strachan AF, Van Der Westhuyzen DR (1986). Serum amyloid A-containing human high density lipoprotein 3. Density, size, and apolipoprotein composition. *Journal of Biological Chemistry*.

[9] Uhlar CM, Whitehead AS (1999). Serum amyloid A, the major vertebrate acute-phase reactant. *European Journal of Biochemistry*.

[10] Sipe J (1999). Part 2: Revised nomenclature for serum amyloid a (SAA). *Amyloid*.

[11] Whitehead AS, De Beer MC, Steel DM (1992). Identification of novel members of the serum amyloid A protein superfamily as constitutive apolipoproteins of high density lipoprotein. *Journal of Biological Chemistry*.

[12] Chiba T, Han CY, Valsar T (2009). Serum amyloid A3 does not contribute to circulating SAA levels. *Journal of Lipid Research*.

[13] De Beer MC, Webb NR, Wroblewski JM (2010). Impact of serum amyloid A on high density lipoprotein composition and levels. *Journal of Lipid Research*.

[14] McGillicuddy FC, de la Llera Moya M, Hinkle CC (2009). Inflammation impairs reverse cholesterol transport in vivo. *Circulation*.

[15] Annema W, Nijstad N, Tölle M (2010). Myeloperoxidase and serum amyloid A contribute to impaired in vivo reverse cholesterol transport during the acute phase response but not group IIA secretory phospholipase A2. *Journal of Lipid Research*.

[16] Malik P, Berisha SZ, Santore J, Agatisa-Boyle C, Brubaker G, Smith JD (2011). Zymosan-mediated inflammation impairs in vivo reverse cholesterol transport. *Journal of Lipid Research*.

[17] Yona S, Heinsbroek SEM, Peiser L, Gordon S, Perretti M, Flower RJ (2006). Impaired phagocytic mechanism in annexin 1 null macrophages. *British Journal of Pharmacology*.

[18] Zhao Z, De Beer MC, Cai L (2005). Low-density lipoprotein from apolipoprotein E-deficient mice induces macrophage lipid accumulation in a CD36 and scavenger receptor class A-dependent manner. *Arteriosclerosis, Thrombosis, and Vascular Biology*.

[19] Zhang Y, Zanotti I, Reilly MP, Glick JM, Rothblat GH, Rader DJ (2003). Overexpression of apolipoprotein A-I promotes reverse transport of cholesterol from macrophages to feces in vivo. *Circulation*.

[20] Asztalos BF, De La Llera-Moya M, Dallal GE, Horvath KV, Schaefer EJ, Rothblat GH (2005). Differential effects of HDL subpopulations on cellular ABCA1- and SR-BI-mediated cholesterol efflux. *Journal of Lipid Research*.

[21] Francis GA, Knopp RH, Oram JF (1995). Defective removal of cellular cholesterol and phospholipids by apolipoprotein A-I in Tangier disease. *Journal of Clinical Investigation*.

[22] Alexander ET, Vedhachalam C, Sankaranarayanan S (2011). Influence of apolipoprotein A-I domain structure on macrophage reverse cholesterol transport in mice. *Arteriosclerosis, Thrombosis, and Vascular Biology*.

[23] Sabeva NS, Rouse EJ, Graf GA (2007). Defects in the leptin axis reduce abundance of the ABCG5-ABCG8 sterol transporter in liver. *Journal of Biological Chemistry*.

[24] Webb NR, Connell PM, Graf GA (1998). SR-BII, an isoform of the scavenger receptor BI containing an alternate cytoplasmic tail, mediates lipid transfer between high density lipoprotein and cells. *Journal of Biological Chemistry*.

[25] Alexander ET, Weibel GL, Joshi MR (2009). Macrophage reverse cholesterol transport in mice expressing ApoA-I milano. *Arteriosclerosis, Thrombosis, and Vascular Biology*.

[26] Nieland TJF, Penman M, Dori L, Krieger M, Kirchhausen T (2002). Discovery of chemical inhibitors of the selective transfer of lipids mediated by the HDL receptor SR-BI. *Proceedings of the National Academy of Sciences of the United States of America*.

[27] Hayat S, Raynes JG (1997). Serum amyloid A has little effect on hight density lipoprotein (HDL) binding to U937 monocytes but may influence HDL mediated cholesterol transfer. *Biochemical Society Transactions*.

[28] Tam SP, Flexman A, Hulme J, Kisilevsky R (2002). Promoting export of macrophage cholesterol: the physiological role of a major acute-phase protein, serum amyloid A 2.1. *Journal of Lipid Research*.

[29] Stonik JA, Remaley AT, Demosky SJ, Neufeld EB, Bocharov A, Brewer HB (2004). Serum Amyloid a promotes ABCA1-dependent and ABCA1-independent lipid efflux from cells. *Biochemical and Biophysical Research Communications*.

[30] Van Der Westhuyzen DR, Cai L, De Beer MC, De Beer FC (2005). Serum amyloid A promotes cholesterol efflux mediated by scavenger receptor B-I. *Journal of Biological Chemistry*.

[31] Abe-Dohmae S, Kato KH, Kumon Y (2006). Serum amyloid A generates high density lipoprotein with cellular lipid in an ABCA1- or ABCA7-dependent manner. *Journal of Lipid Research*.

[32] Marsche G, Frank S, Raynes JG, Kozarsky KF, Sattler W, Malle E (2007). The lipidation status of acute-phase protein serum amyloid A determines cholesterol mobilization via scavenger receptor class B, type I. *Biochemical Journal*.

[33] De Beer MC, Ji A, Jahangiri A (2011). ATP binding cassette G1-dependent cholesterol efflux during inflammation. *Journal of Lipid Research*.

[34] Cai L, De Beer MC, De Beer FC, Van Der Westhuyzen DR (2005). Serum amyloid a is a ligand for scavenger receptor class B type I and inhibits high density lipoprotein binding and selective lipid uptake. *Journal of Biological Chemistry*.

[35] Brown JM, Bell TA, Alger HM (2008). Targeted depletion of hepatic ACAT2-driven cholesterol esterification reveals a non-biliary route for fecal neutral sterol loss. *Journal of Biological Chemistry*.

[36] Kruit JK, Plösch T, Havinga R (2005). Increased fecal neutral sterol loss upon liver X receptor activation is independent of biliary sterol secretion in mice. *Gastroenterology*.

[37] van der Veen JN, van Dijk TH, Vrins CLJ (2009). Activation of the liver X receptor stimulates trans-intestinal excretion of plasma cholesterol. *Journal of Biological Chemistry*.

[38] van der Velde AE, Vrins CLJ, van den Oever K (2007). Direct intestinal cholesterol secretion contributes significantly to total fecal neutral sterol excretion in mice. *Gastroenterology*.

[39] Temel RE, Sawyer JK, Yu L (2010). Biliary sterol secretion is not required for macrophage reverse cholesterol transport. *Cell Metabolism*.

[40] Nijstad N, Gautier T, Briand F, Rader DJ, Tietge UJF (2011). Biliary sterol secretion is required for functional in vivo reverse cholesterol transport in mice. *Gastroenterology*.

